# Long-term antimicrobial assessment of orthodontic brackets coated with nitrogen-doped titanium dioxide against *Streptococcus mutans*

**DOI:** 10.1186/s40510-018-0236-y

**Published:** 2018-09-17

**Authors:** Parisa Salehi, Neda Babanouri, Mehdi Roein-Peikar, Fatemeh Zare

**Affiliations:** 1 Orthodontic Research Center, School of Dentistry, Shiraz University of Medical Sciences, Qom Abad Blv, Ghasrodasht, Shiraz, AV Iran; 2 Johns Hopkins University of Medicine, Baltimore, USA; 3 Shiraz, Iran

**Keywords:** Photocatalytic TiO2, Bracket, Antimicrobial activity, Streptococcus mutans

## Abstract

**Background:**

The antimicrobial properties of orthodontic wire and brackets with nitrogen-doped titanium dioxide (N-doped TiO2) coating have been studied in the past. However, the evaluation period had been short and limited to 30 days. The aim of the present study was to extend the evaluation period (up to 90 days) of assessing the long-term antimicrobial effects of stainless steel orthodontic brackets coated with nitrogen-doped titanium dioxide (N-doped TiO2).

**Methods:**

A total of 40 stainless steel pre-adjusted premolar brackets were equally divided into two groups; namely the control group (*n*=20, uncoated brackets) and the experimental group (*n*=20, coated brackets). RF magnetron sputtering was used to apply a thin film of TiO2 on the bracket surface. The crystalline structure of the thin film was assessed using X-ray diffraction. The antimicrobial property of the brackets against *Streptococcus mutans* (*S. mutans*) was evaluated using the survival rate by colony-forming units (CFU) at four intervals: 24 hours (T0), 30 days (T1), 60 days (T2), and 90 days (T3). 2-way ANOVA Repeated Measures was used to compare the effects between the groups over the time.

**Results:**

There was no significant interaction between group and time (*p* = 0.568). The orthodontic brackets coated with the N-doped TiO2 thin film showed a significant CFU reduction (37.71 ± 5.21, 37.81 ± 5.03, 37.98 ± 5.37, and 37.74 ± 5.21 at T0, T1, T2, and T3, respectively) compared to the uncoated brackets (400.91 ± 14.67, 401.58 ± 14.01, 400.31 ± 14.68, and 402.04 ± 13.98 at T0, T1, T2, and T3, respectively) through visible light (*p* < 0.001).

**Conclusion:**

N-doped TiO2 coated orthodontic brackets showed strong antimicrobial property against *S. mutans* over a period of 90 days, which is effective in preventing enamel decalcification during orthodontic therapy.

## Background

Fixed orthodontic appliances reduce the self-cleaning capacity of tooth surface and increase the level of cariogenic bacteria such as *Streptococcus mutans* and lactobacilli in saliva and dental plaque. As a result, wearing fixed orthodontic appliances can increase the risk of white spot formation or demineralization [1–3].

Many researchers have evaluated a variety of techniques to prevent the occurrence of white spot lesions during fixed orthodontic treatment. In this regard, various strategies have been suggested (e.g., educating patients about dental plaque management, use of fluoride mouth rinse and dentifrice, use of probiotics and antibiotics, fluoride-releasing adhesive, and professional cleaning). However, these strategies are unsuccessful without active participation and compliance by patients. Note that among the above-mentioned strategies, long-term efficacy of fluoride-containing adhesives requires further investigation [4].

Recently, the antibacterial properties of semiconductor nanoparticles such as titanium dioxide (TiO2), zinc oxide, tungsten oxide, and iron oxide have attracted great attention [5]. Nanoparticles are viewed as insoluble nanomaterials with a size smaller than 100 nm [6]. In comparison with non-nanoscale particles, they have a greater surface-to-volume ratio (per unit mass), interact more closely with microbial membranes, and provide a substantially larger surface area for antimicrobial activity [7]. Many bacterial strains are becoming antibiotic-resistant; however, they are less likely to develop resistance against metal nanoparticles than conventional antibiotics [8]. Hence, there is a renewed interest to resort to alternative antibacterial agents among which metal nanoparticles (in the range of 1–10 nm in size) are of particular interest due to their high biocidal activity against bacteria [7].

Among these nanoparticles, TiO2 has been widely applied in organic degradation processes due to its biocompatibility and chemical stability [9–11]. Upon illumination by ultraviolet light (< 385 nm wavelength), TiO2 becomes strongly oxidative. In the valence band, it generates holes and hydroxyl radicals, whereas, in the conduction band, electrons and superoxide ions are generated. Moreover, the photocatalytic reaction (i.e., oxidation from organics to carbon dioxide) can decompose organic compounds [12]. By doping and surface modification, TiO2 exhibits catalytic activity in the visible light region and increases the utilization rate of visible light. Consequently, it leads to the photocatalytic efficiency of nano-TiO2 [13].

To benefit from the antibacterial properties of nanoparticles, two main strategies are adopted in orthodontics to reduce biofilm formation. One strategy is to combine orthodontic adhesives or acrylic materials with nanoparticles. The alternative is coating the surface of orthodontic brackets or wires with nanoparticles [7, 14]. Short-term studies have demonstrated that by incorporating TiO2 or silver nanoparticles into orthodontic adhesives, the antibacterial activity is significantly enhanced without compromising mechanical properties (e.g., shear bond strength) [15, 16].

Antimicrobial performance of orthodontic wires and brackets coated with photocatalytic TiO2 has been evaluated in the previous studies [5, 13, 17–19]. Cao et al. investigated the antibacterial and anti-adherent properties of orthodontic brackets coated with a thin layer of N-doped TiO2 against *Streptococcus mutans*, *Lactobacillus acidophilus*, *Actinomyces viscous*, and *Candida albicans* through visible light. They reported that the brackets coated with the N-doped TiO2-xNy thin film showed high antimicrobial and bacterial adhesive properties against normal oral pathogenic bacteria [13].

Although the use of orthodontic brackets or wires coated with nanoparticles have been investigated previously, review of current literature on orthodontic material showed that the evaluation period had been short and limited to 24 h or a few weeks, while a comprehensive orthodontic treatment usually lasts more than 1.5 years [14]. Moreover, TiO2 coating of orthodontic appliances is an expensive and a time-consuming process. For this strategy to be both successful and cost-beneficial, an investigation on the long-term antimicrobial effect of TiO2 bracket is essential. Since *S. mutans* is the main cause of tooth decay initiation and progression, [20] the present study aimed to evaluate the long-term (90 days) antimicrobial properties of the stainless steel orthodontic brackets coated with N-doped TiO2 thin film against *S. mutans*.

## Methods

The present study was performed on 40 stainless steel pre-adjusted premolar brackets (Dentaurum; Ispringen, Germany). The specimens were equally divided into two groups, namely, the control group (*n* = 20, uncoated brackets) and the experimental group (*n* = 20, N-doped TiO2 coated brackets).

### Preparation of N-doped TiO2 thin film

The brackets were ultrasonicated in 99% ethanol and 99.5% acetone for 30 min, in each, to remove macroscopic contaminants and then dried in a desiccator. The photocatalytic TiO2 thin-layer was decomposed on the brackets using the radiofrequency (RF) magnetron sputtering technique.

In the sputtering process, surface atoms or molecules are removed from a solid cathode (target) by bombarding the target with positive ions from an inert gas discharge and deposited on the nearby substrate to form a thin layer. In the present study, substrates were pumped down in a vacuum chamber to the prescribed process pressure of 3.0 × 10^− 3^ Pa. Prior to the coating process, the target was pre-sputtered for 10 min in order to remove any pollutants. The bracket (as substrate) and TiO2 with 99.99% purity (as a target) were placed in the magnetron sputtering chamber. High purity argon was used as the ambient gas. The attachment side of the brackets was not sputtered. The sputtering was conducted for 4 h, the brackets were cooled to room temperature and, subsequently, annealed in nitrogen gas at 450 °C for 2 h.

### Characterization

To investigate anatase phase formation, X-ray diffraction (XRD) with CuKα radiation was used by an X-ray diffractometer (D8, Bruker) after the coating procedure.

### Antibacterial assay of orthodontic brackets

*S. mutans* (ATCC 25175) and TPY agar (ADSA, Barcelona, Spain) were selected for the antibacterial assay. The colony counting method was used in the present experiment. A suspension of *S. mutans* was prepared at a concentration of 1.5 × 10^6^ colony forming units (CFU)/mL. Then, 100 μL of the suspension was added to 3 mL of liquid medium in tubes. The experimental group (TiO2 coated brackets) and the control group (uncoated brackets) were transferred in the tubes. The tubes were incubated at 37 °C for 60 min under visible light with the intensity of 100 W*2. After illumination, 10 μL of the suspension was diluted in 10 mL of sterile saline, and then 10 μL of this dilution was plated onto TPY agar plates and cultured at 37 °C for 24 h. Antibacterial activity was evaluated by colony counting on each plate (T0). This procedure was repeated at 30 days (T1), 60 days (T2), and 90 days (T3) intervals. After each process, the brackets were cleaned in phosphate-buffered saline and immersed in artificial saliva at 37 °C in an incubator; the artificial saliva was replenished every 48 h. The brackets were also subjected to sessions of 1000 thermocycles (1-min duration) from 5 °C to 55 °C to simulate oral conditions.

### Statistical analysis

The data were analyzed using the SPSS software, version 11.0 (statistical package for social sciences, Chicago, Illinois, USA). Descriptive statistical analysis of data was performed for colony counting in both groups at all time intervals. The data were presented as mean ± SD. The normal distribution of the data was tested with the Kolmogorov-Smirnov normality test prior to the application of parametric tests. The two-way ANOVA repeated measures approach was used to compare the effects between the groups over time. *p* < 0.05 were considered statistically significant.

## Results

### X-ray diffractometer

The X-ray diffraction results characterized the crystalline microstructures of the TiO2 thin layers. Thin films which were annealed at 450 °C for 2 h clearly showed two major peaks at 2*θ* = 25.3° and 37.6° attributed to the (101) plane of the anatase phase and (004) plane to the sign of rutile phase, respectively.

### Antibacterial activity of N-doped TiO2 coated brackets against *S. Mutans*

**Fig. 1:**
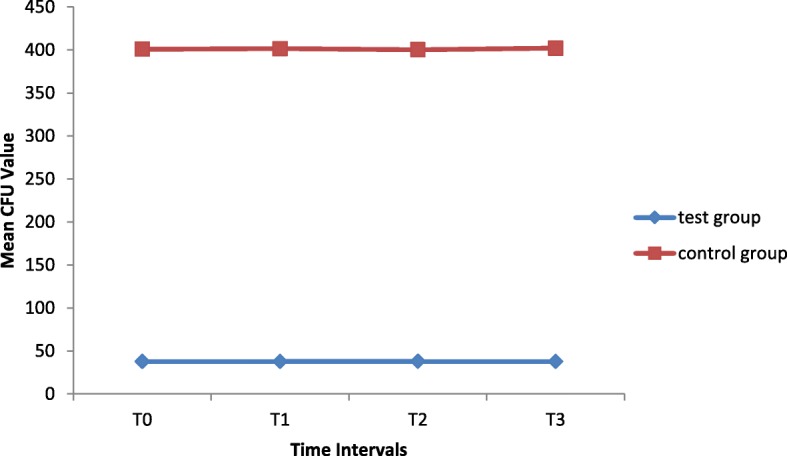
Colony count in the control group (normal stainless steel bracket) and test group (bracket coated with the N-doped TiO2). T0 (24 h), T1 (30 days), T2 (60 days), T3 (90 days)

The survival rate of *S.mutans* was evaluated in terms of CFUs. Table 1 outlines mean ± SD of CFU for the two study groups at time points. The survival rate of *S.mutans* was 37.71 ± 5.21 in the experimental group and 400.91 ± 14.67 in the control group at 24 h after the coating procedure. There was no significant interaction effect between the groups and time (*p* = 0.598). Therefore, the results of main effects for time and group were reported. By controlling the effect of time, the overall mean CFU of the nano-TiO2 coated brackets (37.82 ± 5.15) was significantly lower than the control group (401.21 ± 13.72) (*p* < 0.001).However, the mean CFU values at different time intervals were not statistically different when the effect of group was adjusted. (*p* = 0.696).TiO2 coated brackets showed consistently more antibacterial effect during the entire study period and this property did not decrease over time (Fig. 1).

**Table 1:** Means, standard deviation and adjusted mean of colony count for two study groups

	T0	T1	T2	T3	Adjusted mean
Test group	37.71 ± 5.21	37.81 ± 5.03	37.98 ± 5.37	37.74 ± 5.21	37.82 ± 13.75
Control Group	400.91 ± 14.67	401.58 ± 14.01	400.31 ± 14.68	402.04 ± 13.98	401 ± 5.15
Adjusted Mean	219.31 ± 184.23	219.700 ± 184.49	219.47 ± 183.79	219.89 ± 184.76	219.51 ± 184.29

T0 (24 h), T1 (30 days), T2 (60 days), T3 (90 days)

## Discussion

In the present study, the antimicrobial effect of N-doped TiO2 thin film brackets was evaluated and compared to the uncoated stainless steel brackets during a period of 90 days using survival rate by colony-forming units of *S. mutans*. TiO2 is an inorganic photocatalytic agent with a wide range of antimicrobial activity. The photocatalytic activity of the irradiated TiO2 has been utilized in several areas such as the decomposition of air pollutants, water-treatment procedure, self-cleaning glass, and antibacterial tiles [21, 22]. TiO2 shows strong oxidation activity when illuminated with ultraviolet (UV) light at wavelengths less than 385 nm. Illuminated TiO2 generates excess electrons (e^−^) in the conduction band and positive holes (h^+^) in the valence band, which react with the H_2_O molecules to respectively produce superoxide ions and hydroxyl radicals [13, 23]. These reactive oxygen species are strongly oxidant and react with biologic molecules such as lipids, proteins, and nucleic acid, which induce oxidative damage to the cell membrane and bacterial death [24, 25].

The benefits of antimicrobial property of illuminated TiO2 have been investigated in previous studies. Chun et al. and Shah et al. coated a layer of TiO2 over stainless steel wire and brackets. They reported a significant reduction in *S. mutans, P. gingivalis,* and *L. acidophilus* count [17]. Özyildiz et al. evaluated the antimicrobial effect of TiO2-coated ceramic brackets and reported an effective reduction in the populations of *S.mutans* and *C. albicans* compared to the uncoated brackets [26]. However, the TiO2 absorption band is confined within the UV region (< 380 nm). It only can utilize UV light that accounts for 5% of the sunlight. In practical applications, one should not disregard the photochemical hazard of the UV light source to the skin and eyes of both the patients and dental staff. By doping and surface modification, TiO2 exhibits catalytic activity in the visible light region and increases the utilization rate of visible light. Consequently, it leads to the photocatalytic efficiency of nano-TiO2 [13]. N-doping is shown to be the supreme method. It is also found that TiO2 modified with N-doping has exceptional visible light and UV light activities [27]. Asahi advocated that N-doping would result in a narrowing of the band gap and red-shift of absorption band through a combination of N2p and O2p stages [27].

Cao et al. sputtered a thin film of N-doped TiO2 over stainless steel brackets and reported 95.19%, 91.00%, 69.44%, and 98.86% antimicrobial activity against *S. mutans, L. Acidophilus, A. viscous,* and *C. albicans,* respectively [13]. However, in their study, coated brackets were irradiated under visible light for 24 h, which seems infeasible in clinical situations. They evaluated the antibacterial performance after coating and illumination procedures at one interval only. However, for this strategy to be successful in clinical conditions, the long-term antimicrobial efficacy of TiO2 coated bracket should be investigated and proven.

In the present study, the exposure time was reduced to 60 min which would be more feasible in clinical practice. Additionally, the antimicrobial performance of N-doped TiO2 coated brackets was investigated during a period of 90 days. The results showed that the colony count of *S. mutans* in N-doped TiO2 coated brackets was significantly lower than the uncoated control group at T0 (24 h after coating). This finding is in agreement with those reported in previous studies [13, 17, 26]. It also implies that a reduction in the illumination time did not have any adverse effect on the antimicrobial property of the illuminated TiO2. In the present study, N-doped TiO2 coated brackets maintained their significant antimicrobial property at T1 (31 days), T2 (61 days), and T3 (91 days) after coating procedures compared to the control group. To the best of our knowledge, no other studies have assessed the long-term antimicrobial efficacy of N-doped TiO2 coated brackets. Hence, a comparison with our results could not be performed. Poosti et al. blended light cure orthodontic composite resin with the nanoparticle of TiO2. They reported a significant antimicrobial activity instantly and 30 days after curing [15].

Several techniques have been introduced to prepare TiO2 membrane. Recently, the RF magnetron sputtering deposition, as used in the present study, has been proposed by many researchers due to its numerous desirable characteristics (e.g., ease of sputtering any metal alloy or compound, low deposition temperature, the formation of high purity film, ability to form a uniform and dense coating, and strong coating adhesion) [13, 28, 29].

The antibacterial efficacy of TiO2 is directly related to the photocatalytic activity, and its photocatalytic performance is influenced by the crystalline structure of TiO2 [30]. TiO2 may be found in one of the following crystalline structures: brookite, rutile, and anatase. Brookite is an amorphous type which transforms to the anatase or rutile in both low and high temperatures. It has been shown that rutile is more thermodynamically stable than anatase; whereas, anatase has a higher photocatalytic activity [31, 32]. In the present study, XRD was used to analyze the crystalline structure of sputtered TiO2. The XRD evaluation of TiO2 film deposited on the stainless steel brackets revealed a peak of anatase formation, which is ideal for photocatalytic activity.

The biocompatibility of photocatalytic TiO2 is the essential and prerequisite condition before it can be applied in clinical practice. Historically, titanium is known as an inert, biocompatible, and corrosion resistant metal.

Photocatalytic TiO2 releases hydroxyl radicals (OH°) that decompose organic compound, damages cell walls of microorganisms and may affect normal oral epithelial cells. Although this could be considered as a troubling notion, no harmful effects have been reported. In regard to human periodontal ligament fibroblasts, the proliferation did not decrease on the UV-irradiated TiO2 coated disks [33]. Additionally, in rats exposed to OH°, no abnormal findings were observed in the oral mucosa or the skin [34]. In animal experiments, it is also shown that the TiO2-xNy thin film displays high biocompatibility and the coated bracket with the film does not instigate mucosa irritation, short-term systemic toxicity, hemolysis, or genetic toxicity [13]. TiO2 nanoparticles, as an ingredient, are extensively used in commercial products such as toothpaste, sunscreens, and foods [35]. There are no reports of serious detrimental effects from such products. Nonetheless, further research is required to investigate the aspect of corrosion on the bracket (coated with the N-doped TiO2-xNy thin film) in the oral micro-environment. Also, the toxicity of the corrosion compound on the human body should be assessed.

## Conclusion

Within the limits of this in vitro study, it was concluded that surface modification of stainless steel brackets with N-doped TiO2 thin film can prevent the growth of *S. mutans* at least for 3 months. Reduction of illumination time from 24 h to 60 min did not have any adverse effect on the antibacterial property of N-doped TiO2 coated brackets. Further clinical studies are required to investigate the antimicrobial and anti-caries properties of TiO2 coated brackets as well as its safety aspect during orthodontic treatments.

## References

[1] Rosenbloom RG, Tinanoff N (1991). Salivary Streptococcus mutans levels in patients before, during, and after orthodontic treatment. Am J Orthod Dentofac Orthop.

[2] Jordan C1, LeBlanc DJ. Influences of orthodontic appliances on oral populations of mutans streptococci. Oral Microbiol Immunol. 2002;17(2):65–71. 10.1046/j.0902-0055.2001.00083.x 11929551

[3] Derks A, Katsaros C, Frencken JE, vant’Hof MA, Kuijpers-Jagtman AM (2004). Caries-inhibiting effect of preventive measures during orthodontic treatment with fixed appliances. Caries Res.

[4] Bishara SE, Ostby AW (2018). White spot lesions: formation, prevention, and treatment. Semin Orthod.

[5] Shah AG, Shetty PC, Ramachandra CS, Bhat NS, Laxmikanth SM (2011). In vitro assessment of photocatalytic titanium oxide surface modified stainless steel orthodontic brackets for antiadherent and antibacterial properties against lactobacillus acidophilus. Angle Orthod.

[6] Weir E, Lawlor A, Whelan A, Regan F (2008). The use of nanoparticles in anti-microbial materials and their characterization. Analyst.

[7] Allaker RP (2010). The use of nanoparticles to control oral biofilm formation. J Dent Res.

[8] Pal S, Tak YK, Song JM (2007). Does the antibacterial activity of silver nanoparticles depend on the shape of the nanoparticle? A study of the gram-negative bacterium Escherichia coli. Appl Environ Microbiol.

[9] Mattsson A, Lejon C, Bakardjieva S, Stengl V, Österlund L (2013). Characterisation, phase-stability and surface chemical properties of photocatalytic active Zr and Y co-doped anatase TiO2 nanoparticles. J Solid State Chem.

[10] Cui C, Liu H, Li Y, Sun J, Wang R, Liu S (2005). Fabrication and biocompatibility of nano-TiO2/titanium alloys biomaterials. Mater Lett.

[11] Poma AM, Di Bucchianico S, Galano A, Santucci S (2010). Biocompatibility evaluation of TiO2 nanoparticles and thin films by means of the murine macrophages RAW 264.7 cell line. J Biotechnol.

[12] Liang Z, Deng J, Zhao Y, Liu W, Lin An FC (2009). Preparation and characterization of N–I co-doped nanocrystal anatase TiO2 with enhanced photocatalytic activity under visible-light irradiation. Mater Chem Phys.

[13] Cao B, Wang Y, Li N, Liu B, Zhang Y (2013). Preparation of an orthodontic bracket coated with an nitrogen-doped TiO(2-x)N(y) thin film and examination of its antimicrobial performance. Dent Mater J.

[14] Borzabadi-Farahani A, Borzabadi E, Lynch E (2014). Nanoparticles in orthodontics, a review of antimicrobial and anti-caries applications. Acta Odontol Scand.

[15] Poosti M, Ramazanzadeh B, Zebarjad M, Javadzadeh P, Naderinasab M, Shakeri MT (2013). Shear bond strength and antibacterial effects of orthodontic composite containing TiO2 nanoparticles. Eur J Orthod.

[16] Ahn S-J, Lee S-J, Kook J-K, Lim B-S (2009). Experimental antimicrobial orthodontic adhesives using nanofillers and silver nanoparticles. Dent Mater.

[17] Chun MJ, Shim E, Kho EH, Park KJ, Jung J, Kim JM (2007). Surface modification of orthodontic wires with photocatalytic titanium oxide for its antiadherent and antibacterial properties. Angle Orthod.

[18] Cao S, Wang Y, Cao L, Wang Y, Lin B, Lan W (2016). Preparation and antimicrobial assay of ceramic brackets coated with TiO2thin films. Korean J Orthod.

[19] Ghasemi T, Arash V, Rabiee SM, Rajabnia R, Pourzare A, Rakhshan V (2017). Antimicrobial effect, frictional resistance, and surface roughness of stainless steel orthodontic brackets coated with nanofilms of silver and titanium oxide: a preliminary study. Microsc Res Tech.

[20] Loesche WJ (1986). Role of Streptococcus mutans in human dental decay. Microbiol Rev.

[21] Foster HA, Ditta IB, Varghnese S (2011). Photocatalytic disinfection using titanium dioxide: spectrum and mechanism of antimicrobial activity. Appl Microbiol Biotechnol.

[22] Lakshmi S, Renganathan R, Fujita S (1995). Study on TiO2-mediated photocatalytic degradation of methylene blue. J Photochem Photobiol A Chem.

[23] Priya R, Baiju KV, Shukla S, Biju S, Reddy ML, Patil KR, Warrier KG (2009). Enhanced solar-radiation induced photocatalytic activity of surface-modified nanocrystalline anatase-titania. Catal Letters.

[24] Kühn KP, Chaberny IF, Massholder K, Stickler M, Benz VW, Sonntag H-G (2003). Disinfection of surfaces by photocatalytic oxidation with titanium dioxide and UVA light. Chemosphere.

[25] Lee M-C, Yoshino F, Shoji H, Takahashi S, Todoki K, Shimada S (2005). Characterization by electron spin resonance spectroscopy of reactive oxygen species generated by titanium dioxide and hydrogen peroxide. J Dent Res.

[26] Özyildiz F, Güden M, Uzel A, Karaboz I, Akil O, Bulut H (2010). Antimicrobial activity of TiO2-coated orthodontic ceramic brackets against Streptococcus mutans and Candida albicans. Biotechnol Bioprocess Eng.

[27] Asahi R, Morikawa T, Ohwaki T, Aoki K, Taga Y (2001). Visible-light photocatalysis in nitrogen-doped titanium oxides. Science (80- ).

[28] Swann S (1988). Magnetron sputtering. Phys Technol.

[29] Majeed A, He J, Jiao L, Zhong X, Sheng Z (2015). Surface properties and biocompatibility of nanostructured TiO(2) film deposited by RF magnetron sputtering. Nanoscale Res Lett.

[30] Singh P, Kaur D (2010). Room temperature growth of nanocrystalline anatase TiO2 thin films by dc magnetron sputtering. Phys B Condens Matter.

[31] Horiuchi Y, Horiuchi M, Hanawa T, Soma K (2007). Effect of surface modification on the photocatalysis of Ti-Ni alloy in orthodontics. Dent Mater J.

[32] Hurum DC, Agrios AG, Gray KA, Rajh T, Thurnauer MC (2003). Explaining the enhanced photocatalytic activity of Degussa P25 mixed-phase TiO2 using EPR. J Phys Chem B.

[33] SHIRAI R, MIURA T, YOSHIDA A, YOSHINO F, ITO T, YOSHINARI M (2016). Antimicrobial effect of titanium dioxide after ultraviolet irradiation against periodontal pathogen. Dent Mater J.

[34] Yamada Y, Mokudai T, Nakamura K, Hayashi E, Kawana Y, Kanno T (2012). Topical treatment of oral cavity and wounded skin with a new disinfection system utilizing photolysis of hydrogen peroxide in rats. J Toxicol Sci.

[35] Weir A, Westerhoff P, Fabricius L, Hristovski K, von Goetz N (2012). Titanium dioxide nanoparticles in food and personal care products. Environ Sci Technol.

